# Adherence to recombinant human growth hormone therapy in children: influencing factors and clinical implications

**DOI:** 10.3389/fped.2025.1617579

**Published:** 2025-09-02

**Authors:** Wenjing Chang, Hua Jin, Qin Zhou, Kan Ye, Yuee Dai

**Affiliations:** ^1^ Department of Child Health Care, The Affiliated Suzhou Hospital of Nanjing Medical University, Gusu School, Nanjing Medical University, Suzhou, China; ^2^ Department of Child Health Care, Suzhou Women and Children Health Hospital, The Affiliated Suzhou Hospital of Nanjing Medical University, Suzhou, China; ^3^ Department of Child Health Care, The Affiliated Suzhou Hospital of Nanjing Medical University, Suzhou Women and Children Health Hospital, Suzhou, China

**Keywords:** recombinant human growth hormone, adherence, influencing factors, pediatric patients, growth disorders

## Abstract

**Background:**

Adherence to recombinant human growth hormone (rhGH) therapy is crucial for achieving optimal outcomes in children with growth disorders. However, non-adherence remains a significant challenge, affecting treatment efficacy and patient prognosis. This study evaluates adherence rates to rhGH therapy in pediatric patients, identifies key influencing factors, and explores strategies to improve adherence.

**Methods:**

A retrospective analysis was conducted on 8,621 pediatric patients receiving rhGH therapy in China. Adherence was assessed by the proportion of prescribed doses taken, with good adherence defined as ≥86%. Factors influencing adherence were analyzed using logistic regression models, considering variables such as age, GH formulation type, treatment duration, and regional differences.

**Results:**

The overall mean adherence rate was 92%, with long-acting GH formulations associated with significantly higher adherence than daily GH injections (94% vs. 91%, *p* < 0.001). Older children (12–18 years) exhibited better adherence than younger age groups. Patients with severe growth deficits (≤P3 percentile) showed higher adherence than those with moderate deficits. Longer treatment duration was linked to decreased adherence. Regional differences were observed, with patients from Northern Jiangsu demonstrating better adherence than those from Southern Jiangsu.

**Conclusion:**

Adherence to rhGH therapy is influenced by multiple factors, including GH formulation, age, severity of growth deficit, treatment duration, and regional disparities. Long-acting GH formulations significantly improve adherence. Tailored interventions, such as parental education, digital adherence monitoring, and personalized support programs, are essential to sustain long-term adherence and optimize treatment outcomes.

## 1 Introduction

Growth hormone (GH) is a 191-amino acid peptide secreted by the anterior pituitary that stimulates metabolism and promotes growth and maturation of tissues, organs, and bones, playing a key role in overall development (1). GH deficiency or dysregulation can lead to impaired growth, delayed puberty, and metabolic disturbances, significantly affecting an individual's physical and psychological well-being (2, 3). Advances in understanding GH's amino acid sequence and molecular structure, along with progress in genetic engineering, have led to the widespread use of recombinant human growth hormone (rhGH) since the 1980s (4). Currently, rhGH is extensively applied in the treatment of growth disorders, including idiopathic short stature (ISS), short stature in children born small for gestational age (SGA), Turner syndrome–associated short stature, and GH deficiency (GHD) in both children and adults (5–7).

Given the nature of GH therapy, long-term treatment is required to achieve the desired therapeutic outcomes. Clinical studies have demonstrated that adherence to GH therapy is a critical determinant of treatment success (8). Poor adherence has been associated with suboptimal growth responses, reduced final adult height, and increased treatment burden (8, 9). Rosenfeld and Bakker found that children with poor adherence to GH therapy had a significantly lower annual height velocity than those with good adherence, leading to reduced overall growth potential (10). Similarly, a large cohort study by Cutfield et al. revealed that children who missed more than 20% of their prescribed GH doses exhibited diminished growth responses, with height standard deviation (SD) scores significantly lower than those who adhered to treatment (11). These findings highlight the necessity of adherence to GH therapy in achieving optimal therapeutic benefits.

Adherence refers to the extent to which an individual's behavior aligns with clinical prescriptions or medical advice, specifically the degree to which a patient follows the prescribed medication regimen provided by healthcare providers. Non-adherence remains a major challenge in GH therapy, with behaviors ranging from occasionally missing a single dose to taking a reduced dosage or even completely discontinuing medication. A systematic review reported that adherence to GH therapy varies widely from as low as 5% to as high as 82% (12). Factors influencing adherence include treatment burden, injection-related anxiety, lack of perceived benefits, socioeconomic status, and parental involvement in medication administration (12).

Despite the well-documented impact of adherence on GH therapy outcomes in Western populations, there is a lack of comprehensive research on adherence to GH therapy among children in China. Differences in healthcare accessibility, parental awareness, and cultural perceptions of growth disorders may lead to variations in adherence patterns. Therefore, it is crucial to investigate the adherence rates and associated influencing factors in Chinese pediatric patients undergoing rhGH therapy. Understanding these factors is essential for optimizing clinical management strategies, improving adherence, and ultimately enhancing treatment outcomes.

Thus, this study systematically investigates adherence to rhGH therapy in pediatric patients, analyzes the key factors affecting medication adherence, and provides a foundation for developing evidence-based strategies to improve adherence in clinical practice.

## 2 Methods

### 2.1 Study design and patient population

This retrospective study analyzed the factors influencing rhGH treatment adherence in pediatric patients in China. Between February 2012 and August 2024, a total of 8,621 children and adolescents aged 3–18 years were treated with rhGH at health care institutions within Jiangsu Province, China. Each patient's initial visit showed suboptimal height, and after an examination confirmed the conditions for rhGH treatment, a file was established. The patient was then seen regularly every month, with the doctor in charge checking and calculating the dose used, then issuing a quantitative prescription. Medication was checked and recorded at the next visit, with the prescribed dose adjusted accordingly. The percentage adherence for each patient was calculated as the ratio of the dose used to the prescribed dose.

### 2.2 Eligibility criteria

#### 2.2.1 Inclusion criteria

1. Patients: Children at the initiation of rhGH therapy.
2. Indication for rhGH therapy: Diagnosed with growth disorder requiring rhGH treatment, including but not limited to GHD, ISS, TS, and children born small for SGA with persistent short stature.
3. Treatment initiation: Received rhGH therapy at healthcare institutions in Jiangsu Province, China, during the study period. Both long-acting and daily GH used in our cohort were liquid formulations administered via auto-injector pen
4. Regular follow-up: Patients with at least one documented follow-up visit every month for adherence assessment.

#### 2.2.2 Exclusion criteria

1. Patients with missing or incomplete medical records regarding GH dosage, follow-up adherence assessments, or growth response measurements.
2. Concurrent endocrine disorders: Presence of other significant endocrine disorders affecting growth.
3. Chronic systemic diseases: Patients with chronic kidney disease, severe hepatic dysfunction, malignancies, or genetic syndromes other than Turner syndrome that could interfere with GH therapy response.

#### 2.2.3 Ethics

The Ethical Committee of Suzhou Municipal Hospital (Approval K-2023-105-H01) approved the study protocol.

### 2.3 Statistical analysis

Continuous variables were assessed by the Shapiro–Wilk test. Normally distributed variables were expressed as mea*n* ± SD and compared using the two-samples *t*-test or one-way analysis of variance (ANOVA). Non-normally distributed variables were expressed as median (IQR) and compared using the Mann–Whitney *U*-test or Kruskal–Wallis *H*-test. Categorical variables were expressed as frequency (*n*) and percentage (%) and were compared using the chi-square test (χ^2^ test) or Fisher's exact test. All statistical analyses were two-tailed tests, with *p* < 0.05 set as the significance level. Logistic univariate and multivariate analyses were performed to identify independent risk factors affecting rhGH treatment adherence. Multivariate logistic regression was used to analyze the independent risk factors of medication adherence, with *p* < 0.05 considered statistically significant. Statistical analysis and graph plotting were performed using R version 4.3.0 software.

## 3 Results

### 3.1 Patient characteristics

The study included a total of 8,621 patients, with 6,765 receiving daily GH (water-based) injections and 1,856 on long-acting GH formulations. The sex distribution was nearly equal between males (54.0%) and females (46.0%), with no significant difference between the two groups (*p* = 0.962).

The mean age of the study population was 9.06 years (SMD = 0.076), but the patients on long-acting GH were significantly younger (8.89 years vs. 9.11 years, *p* = 0.005). Age was further categorized, with the majority of patients (66.9%) aged 6–12 years, followed by 18.7% in the 3–6 age group. Patients aged 3–6 years were more likely to receive long-acting GH than daily GH (23.7% vs. 17.4%, *p* < 0.001). Most patients were from Southern Jiangsu (hereafter, Sunan) (70.2%), followed by Northern Jiangsu (Subei) (18.4%) and Central Jiangsu (Suzhong) (11.4%), with a significant difference between the two GH groups (*p* < 0.001). The total duration of GH therapy was significantly longer in the daily GH group (mean 417.79 vs. 365.41 days, *p* < 0.001), with more patients in the daily GH group receiving >2 years of treatment (25.3% vs. 14.4%). Detailed information is given in Table 1.

**Table 1:** Descriptive analysis.

Characteristic	Daily GH *N* = 6,765	Long-acting GH *N* = 1,856	Overall *N* = 8,621	SMD^a^	*p*-value
Sex, *n* (%)					
Male	3,653 (54.0%)	1,004 (54.1%)	4,657 (54.0%)	0.002	0.962^b^
Female	3,112 (46.0%)	852 (45.9%)	3,964 (46.0%)		
Age					
*n*	6,765	1,856	8,621	0.076	0.005^c^
Mean (SD)	9.11 (2.71)	8.89 (3.03)	9.06 (2.78)		
Age group, *n* (%)					
3–6 years	1,176 (17.4%)	440 (23.7%)	1,616 (18.7%)	0.193	<0.001^b^
6–12 years	4,655 (68.8%)	1,110 (59.8%)	5,765 (66.9%)		
12–18 years	934 (13.8%)	306 (16.5%)	1,240 (14.4%)		
Region, *n* (%)					
Sunan	4,657 (68.8%)	1,393 (75.1%)	6,050 (70.2%)	0.144	<0.001^b^
Suzhong	795 (11.8%)	191 (10.3%)	986 (11.4%)		
Subei	1,313 (19.4%)	272 (14.7%)	1,585 (18.4%)		
Diagnosed height category, *n* (%)					
<P3	2,302 (38.4%)	612 (35.1%)	2,914 (37.6%)	0.098	0.001^b^
P3-P25	2,327 (38.8%)	666 (38.1%)	2,993 (38.7%)		
>P25	1,368 (22.8%)	468 (26.8%)	1,836 (23.7%)		
Missing	768	110	878		
Total medication duration					
*N*	6,765	1,856	8,621	0.330	<0.001^c^
Mean (SD)	541.79 (417.79)	412.15 (365.41)	513.88 (410.53)		
Total medication duration group, *n* (%)					
<1 year	2,711 (40.1%)	978 (52.7%)	3,689 (42.8%)	0.312	<0.001^b^
1–2 years	2,341 (34.6%)	610 (32.9%)	2,951 (34.2%)		
>2 years	1,713 (25.3%)	268 (14.4%)	1,981 (23.0%)		
Adherence					
*n*	6,765	1,856	8,621	−0.209	<0.001^c^
Mean (SD)	0.91 (0.14)	0.94 (0.12)	0.92 (0.14)		
Adherence ≥80%, *n* (%)					
No	827 (12.2%)	124 (6.7%)	951 (11.0%)	0.190	<0.001^b^
Yes	5,938 (87.8%)	1,732 (93.3%)	7,670 (89.0%)		
Adherence ≥85%, *n* (%)					
No	1,147 (17.0%)	181 (9.8%)	1,328 (15.4%)	0.213	<0.001^b^
Yes	5,618 (83.0%)	1,675 (90.2%)	7,293 (84.6%)		
Adherence ≥90%, *n* (%)					
No	1,693 (25.0%)	311 (16.8%)	2,004 (23.2%)	0.204	<0.001^b^
Yes	5,072 (75.0%)	1,545 (83.2%)	6,617 (76.8%)		

^a^ Standardized mean difference.

^b^ Pearson's Chi-squared test.

^c^ Welch two sample *t*-test.

### 3.2 Adherence analysis

Adherence was assessed as the proportion of prescribed doses taken, with an overall mean adherence of 0.92 (SMD = −0.209) among all patients. Notably, patients receiving long-acting GH exhibited significantly higher adherence than those on daily GH (0.94 vs. 0.91, *p* < 0.001). Additionally, a significantly greater proportion of patients in the long-acting GH group achieved adherence levels of ≥90% (83.2% vs. 75.0%, *p* < 0.001). Similar trends were observed for adherence ≥85% (90.2% vs. 83.0%) and ≥80% (93.3% vs. 87.8%), with all differences reaching statistical significance (*p* < 0.001) (Table 1).

### 3.3 Factors influencing adherence

#### 3.3.1 Overall analysis

Long-acting GH was consistently associated with better adherence (OR 1.573, 95% CI 1.352–1.836, *p* < 0.001). Furthermore, sex had no impact on adherence (OR 1.127, 95% CI 0.999–1.272, *p* = 0.053). In addition, children aged 12–18 years also showed slightly better adherence than younger children (6–12: OR 1.188, 95% CI 1.004–1.403, *p* < 0.001; 12–18: OR 1.607, 95% CI 1.278–2.024, *p* < 0.001) (Table 2).

**Table 2:** Multivariate logistic regression analysis of rhGH medication adherence.

Factors	Category	OR^a^	95% CI^a^	*p*-value
Medication type	Long-acting GH	1.000	–	
	Daily GH	1.573	1.352, 1.836	<0.001
Sex	Male	1.000	–	
	Female	1.127	0.999, 1.272	0.053
Age at first medication (years)	3–6 years	1.000	–	
	6–12 years	1.188	1.004, 1.403	0.044
	12–18 years	1.607	1.278, 2.024	<0.001
Diagnosed height category, *n* (%)	<P3	1.000	–	
	P3–P25	0.808	0.705, 0.927	0.002
	>P25	0.888	0.750, 1.052	0.168
Total medication duration group	<1 year	1.000	–	
	1–2 years	0.644	0.565, 0.733	<0.001
	>2 years	0.703	0.596, 0.829	<0.001
Region	Sunan	1.000	–	
	Suzhong	1.100	0.917, 1.328	0.311
	Subei	1.273	1.088, 1.494	0.003

^a^ OR, odds ratio; CI, confidence interval.

#### 3.3.2 Subgroup analysis stratified by proportion of adherence

##### 3.3.2.1 Adherence >90%

**Figure 1:**
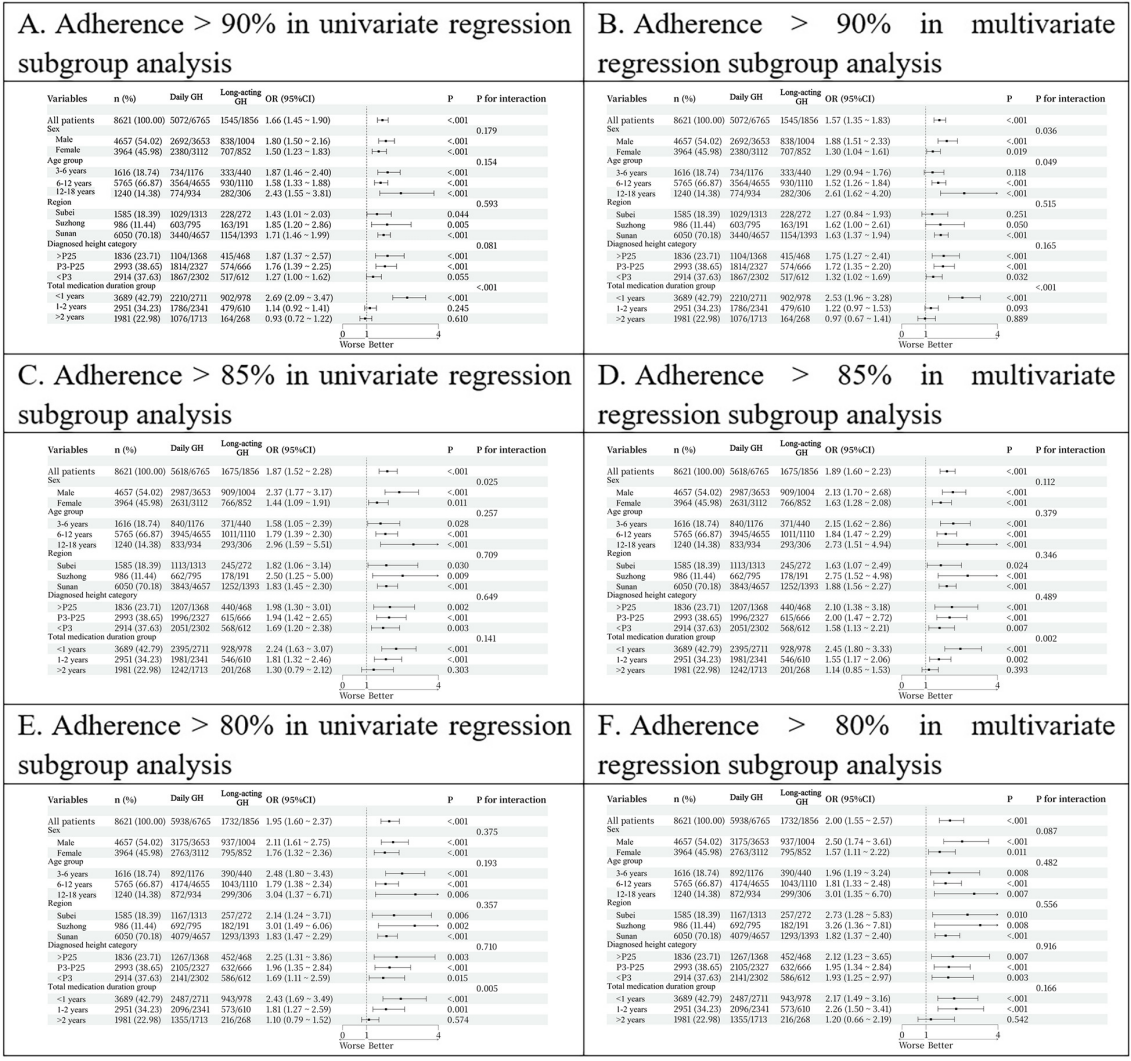
**(A)** adherence >90% in univariate regression subgroup analysis. **(B)** Adherence >90% in multivariate regression subgroup analysis. **(C)** Adherence >80% in univariate regression subgroup analysis. **(D)** Adherence >80% in multivariate regression subgroup analysis.

Univariate regression subgroup analysis indicated that patients in the long-acting GH group demonstrated better adherence than those in the daily GH group, irrespective of gender and age (*p* < 0.001). Regionally, adherence was significantly higher in the long-acting GH group for patients from Sunan (*p* < 0.001) and marginally higher for those from Suzhong (*p* = 0.005), and Subei (*p* = 0.044). Regarding height at diagnosis, long-term adherence was superior in the long-acting GH group for height percentiles > P25 (*p* < 0.001) and P3–P25 (*p* < 0.001) but not for < P3 (*p* = 0.055). Additionally, adherence was higher in the long-acting GH group for patients using the drug for less than one year (*p* < 0.001), whereas no significant difference was noted for durations of one to two years (*p* = 0.610) or beyond two years (*p* = 0.245) (Figure 1A).

Multivariate regression subgroup analysis further confirmed better adherence in the long-acting GH group, specifically in those aged 6–18 years (*p* < 0.001). Regionally, adherence remained superior in Sunan (*p* < 0.001) but not in Subei (*p* = 0.251) or Suzhong (*p* = 0.050). Long-term adherence was significantly higher in the long-acting GH group for patients with height percentiles > P25 (*p* < 0.001) P3–P25 (*p* < 0.001), and < P3 (*p* = 0.032). Adherence was also better in the long-acting GH group for patients using the drug for less than one year (*p* < 0.001), while no differences were observed for one to two years (*p* = 0.093) or beyond two years (*p* = 0.889) (Figure 1B).

##### 3.3.2.2 Adherence >85%

Univariate regression subgroup analysis revealed that patients in the long-acting GH group exhibited significantly better adherence than those in the daily GH group, regardless of gender, age, region, and height at diagnosis (all *p* < 0.05). Additionally, adherence was higher in the long-acting GH group for patients using the drug for less than one year (*p* < 0.001) and one to two years (*p* = 0.002), whereas no significant difference was observed beyond two years (*p* = 0.393) (Figure 1C).

Multivariate regression subgroup analysis further confirmed superior adherence in the long-acting GH group, independent of gender (*p* < 0.05). Moreover, patients aged >3 years demonstrated better adherence with long-acting GH than with daily GH (*p* < 0.05). Regionally, adherence remained superior in Sunan (*p* < 0.001) and Suzhong (*p* = 0.009) and Subei (*p* = 0.030). Long-term adherence was significantly higher in the long-acting GH group across all height percentiles (*p* = 0.002 for > P25, *p* < 0.001 for P3–P25, and *p* = 0.003 for <P3). Additionally, adherence remained superior in the long-acting GH group for patients using the drug for less than one year (*p* < 0.001) and one to two years (*p* < 0.001), with no significant difference beyond two years (*p* = 0.303) (Figure 1D).

##### 3.3.2.3 Adherence >80%

Univariate regression subgroup analysis showed that patients in the long-acting GH group exhibited significantly better adherence than those in the daily GH group, regardless of gender, age, region, and height at diagnosis (all *p* < 0.05). Additionally, adherence was higher in the long-acting GH group for patients using the drug for less than one year (*p* < 0.001) and one to two years (*p* = 0.001), whereas no significant difference was observed beyond two years (*p* = 0.574) (Figure 1E).

Multivariate regression subgroup analysis further confirmed superior adherence in the long-acting GH group, independent of gender (*p* < 0.05). Moreover, patients aged >3 years demonstrated better adherence with long-acting GH than with daily GH (*p* < 0.05). Regionally, adherence remained superior in Sunan (*p* < 0.001), Suzhong (*p* = 0.008), and Subei (*p* = 0.010). Long-term adherence was significantly higher in the long-acting GH group across all height percentiles (*p* = 0.007 for > P25, *p* < 0.001 for P3–P25, and *p* = 0.003 for < P3). Additionally, adherence remained superior in the long-acting GH group for patients using the drug for less than one year (*p* = 0.001) and one to two years (*p* < 0.001), with no significant difference beyond two years (*p* = 0.542) (Figure 1F).

## 4 Discussion

This study provides a comprehensive analysis of adherence to rhGH therapy in pediatric patients and identifies key factors influencing adherence. The findings reveal that long-acting GH formulations are associated with significantly higher adherence rates than daily GH injections, emphasizing the role of treatment burden in patient adherence. Additionally, age, duration of treatment, severity of growth deficit at diagnosis, and regional factors were found to be significant predictors of adherence. But the large series of patients allowed on one hand reliable results, but on the other it obtains results highly significant for minimal differences. One of the most striking findings of this study is the significantly higher adherence rates observed in patients receiving long-acting GH than in those on daily GH therapy. Furthermore, a greater proportion of patients in the long-acting GH group achieved adherence thresholds of ≥90% (83.2% vs. 75.0%), ≥85% (90.2% vs. 83.0%), and ≥80% (93.3% vs. 87.8%), with all differences being statistically significant (*p* < 0.001). These results align with previous studies indicating that treatment convenience is a major determinant of adherence in pediatric patients (12). The reduced injection frequency associated with long-acting GH likely mitigates the psychological and physical burden of daily injections, resulting in better adherence. This suggests that, when clinically appropriate, long-acting GH formulations should be considered as a strategy to improve adherence and optimize treatment outcomes. However, in this study, approximately 75%–80% of the patients were treated less than 2 years, likely due to factors such as achievement of catch-up growth allowing guideline-based discontinuation, economic burden, adverse effects, and broader challenges in GH treatment accessibility and continuity in China which might have an impact on the difference for patients treated with long-acting GH formulation for shorter periods in comparison to once daily therapy.

### 4.1 Age as a determinant of adherence

Our study found that older children (12–18 years) demonstrated better adherence than younger children across all adherence thresholds. This trend may be attributed to greater autonomy, better understanding of treatment benefits, and increased parental reinforcement in older age groups. Conversely, children aged 3–6 years had the lowest adherence rates, which may be due to injection-related anxiety and reliance on parental supervision (13). These findings underscore the need for targeted educational interventions and parental support programs to enhance adherence in younger patients, particularly through strategies such as play therapy, behavioral reinforcement, and digital adherence monitoring. Although some studies revealed that adherence rates were not associated with age (14).

### 4.2 Growth deficit severity and adherence

Interestingly, children diagnosed with moderate height deficits (>P3) exhibited lower adherence rates than those in the most severe category (<P3). This suggests that perceived treatment urgency plays a role in adherence. Parents and patients with milder growth impairments may not perceive GH therapy as critical, leading to inconsistent adherence. These findings highlight the importance of clinician–patient communication in reinforcing the necessity of continued GH therapy regardless of initial severity. Educational efforts should focus on the long-term benefits of sustained GH therapy, even for children with mild-to-moderate growth impairments.

### 4.3 Effect of treatment duration on adherence

Our analysis showed that longer total treatment duration was associated with decreased adherence, with adherence rates declining after the first year of therapy across different adherence thresholds; all *p* < 0.001). This finding is consistent with prior research demonstrating that adherence tends to decline over time, particularly in chronic therapies requiring prolonged commitment (15). Similarly, other study showed that in patients treated for >5 years (*n* = 668), adherence rate declined slightly over the years (16). Potential reasons for this trend include treatment fatigue, reduced perceived efficacy, and parental burnout. This suggests that continuous adherence monitoring and patient engagement strategies are essential throughout GH therapy. Digital reminders, adherence tracking applications, and periodic counseling sessions may help sustain adherence in long-term treatment regimens.

### 4.4 Regional variations in adherence

Significant regional disparities in adherence were observed, with patients from Subei demonstrating higher adherence than those from Sunan at the ≥90% threshold. However, this trend was not consistent at the ≥85% and ≥80% levels. These differences may reflect variations in healthcare accessibility, socioeconomic status, and parental perceptions of treatment. In China, northern regions of Jiangsu such as Subei are generally less developed than southern areas like Sunan, and may have fewer specialized medical resources but higher trust in tertiary care and physician recommendations. In contrast, families in more affluent regions may have greater autonomy in medical decision-making, potentially leading to selective or interrupted treatment. Further studies are warranted to explore these region-specific factors and develop tailored strategies to optimize adherence across different settings.

### 4.5 Clinical implications and future directions

The findings of this study have important clinical implications for optimizing GH therapy in pediatric patients. Given the higher adherence rates associated with long-acting GH formulations, clinicians should consider switching eligible patients from daily GH to long-acting formulations to improve treatment adherence. Furthermore, personalized adherence strategies based on age, treatment duration, and severity of growth impairment should be developed. Younger children may benefit from parental education and psychological support, while adolescents may require self-management tools and mobile health interventions.

It should be noted that the study was conducted in a specialized GH outpatient clinic, which may limit the generalizability of the findings. While such dedicated clinics are not universally implemented across China, most tertiary hospitals have growth and development clinics within departments of pediatrics, child health, or endocrinology, where children with growth disorders including short stature, delayed development, or precocious puberty receive professional assessment and treatment. In our setting, patients were identified and initiated on GH therapy based on guideline defined indications and personal motivation to improve height. The specialized clinic model typically involves a multidisciplinary team and more frequent follow-up, which may contribute to better adherence. However, this structured approach may not fully reflect real-world practice in general outpatient settings, and thus the observed adherence rates may not be directly applicable to less specialized environments. Thus, long-term adherence monitoring programs should be established to mitigate the decline in adherence over time. Digital health technologies, including electronic injection tracking and telemedicine follow-ups, may offer innovative solutions to enhance adherence. Future research should explore intervention strategies tailored to specific patient demographics and regional healthcare settings to further improve adherence outcomes.

### 4.6 Strengths and limitations

This study has several strengths. Firstly, as a multicenter retrospective study incorporating data from multiple medical institutions, it helps minimize selection bias associated with single-center studies and enhances the representativeness and external validity of the results. Secondly, the large sample size allows a more comprehensive and robust analysis of factors influencing GH therapy adherence compared with previous domestic and international studies. Additionally, this study addresses a gap in domestic research on GH therapy adherence by systematically analyzing key influencing factors, thereby offering valuable clinical guidance for developing personalized intervention strategies.

However, this study also has limitations. First, as a retrospective study, the completeness of the data may be limited, with some cases potentially missing or containing incomplete information. Moreover, since data were collected from different centers, variations in treatment protocols and patient follow-up management across institutions might have influenced the results, for instance, height classification data were missing for 10.2% of patients (878 out of 8,621), which may introduce bias. And the absence of efficacy data limits a comprehensive understanding of the impact on treatment adherence which need further research. This missingness could be attributed to incomplete data entry, or situations in which prescriptions were collected by caregivers without a formal clinical assessment of growth status. In future research, we aim to minimize such data gaps through standardized data collection procedures and prospective study design to better control for potential confounding factors. Furthermore, subjective factors that may affect adherence, such as patients' socioeconomic status and psychological conditions, were not examined in depth. In addition, the observed difference between two groups for basic characteristics may also be influenced by the unequal sample sizes between the two groups, which could affect the stability and interpretation of the statistical results. Future prospective studies incorporating surveys or interviews are needed to comprehensively assess the factors influencing GH therapy adherence.

## 5 Conclusion

This study highlights the key determinants of adherence to GH therapy in children, which include treatment formulation, age, severity of growth deficit, treatment duration, and regional factors. The findings emphasize that long-acting GH formulations significantly improve adherence and suggest that interventions tailored to younger children and long-term treatment patients are crucial for sustaining adherence. Understanding these influencing factors is essential for developing targeted strategies to enhance adherence, ultimately improving treatment outcomes and maximizing the benefits of GH therapy in pediatric populations.

## Data Availability

The original contributions presented in the study are included in the article/Supplementary Material, further inquiries can be directed to the corresponding authors.
